# Substance Use among School-Going Adolescents and Young Adults in Rural Mpumalanga Province, South Africa

**DOI:** 10.3390/bs14070543

**Published:** 2024-06-27

**Authors:** Tabeho Godfrey Mmethi, Perpetua Modjadji, Mmampedi Mathibe, Ntevhe Thovhogi, Machoene Derrick Sekgala, Thomas Khomotjo Madiba, Olalekan Ayo-Yusuf

**Affiliations:** 1 Department of Public Health, School of Health Care Sciences, Sefako Makgatho Health Sciences University, Pretoria 0208, South Africa; drmmethi@gmail.com (T.G.M.);; 2 Department of Community Dentistry, School of Dentistry, University of Pretoria, Pretoria 0208, South Africa; 3 Non-Communicable Diseases Research Unit, South African Medical Research Council, Tygerberg, Cape Town 7505, South Africa; 4 Department of Life and Consumer Sciences, College of Agriculture and Environmental Sciences, University of South Africa, Roodepoort, Johannesburg 1709, South Africa; 5 Africa Centre for Tobacco Industry Monitoring and Policy Research (ATIM), School of Health Systems and Public Health, University of Pretoria, Pretoria 0028, South Africa; lekan.ayo-yusuf@up.ac.za

**Keywords:** substance use, adolescents and young adults, high schools, Mpumalanga, South Africa

## Abstract

The ongoing public health crisis of substance use among school adolescents and young adults (AYAs) in South Africa is not new in research parlance, amidst the national policy of drug abuse management in schools. In view of no tangible progress to reduce substance use in high schools in the country, we conducted a cross-sectional quantitative study aimed at investigating substance use among adolescents and young adults in the four public high schools selected through multi-stage sampling in rural Mpumalanga province, South Africa. Data on substance use, demographics, household socio-demographics, and related factors were collected via a validated self-administered questionnaire. Hierarchical logistic regression was performed using STATA 18. The study included 402 AYAs aged between 14 and 23 years (18 ± 1 years), and 45% reported substance use in the last twelve months. Alcohol was the most used substance (74%), followed by cigarettes (12%) and cannabis (11%). AYAs used substances out of social influence, curiosity, to find joy, and to eliminate stress, especially in social events, on the streets, and at home, and reported negative physical health outcomes, mainly hallucinations, sleeping disorders, body weakness, and dry mouths. Hierarchical logistic regression showed that the likelihood of substance use was three times in a particular high school (S4) (AOR = 3.93, 95%CI: 1.72–8.99), twice among the grade 12s (AOR = 2.73, 95%CI: 1.46–5.11), over twenty times in the communities with substance availability (AOR = 22.45, 95%CI: 2.75–183.56), almost ten times among AYAs participating in recreational/sports activities (AOR = 9.74, 95%CI: 4.21–22.52), and twice likely to happen in larger households (AOR = 2.96, 95%CI: 1.57–5.58). Prevention and intervention efforts should consider these specific health concerns to develop targeted strategies for mitigating substance use and its adverse consequences in this vulnerable population towards achieving the United Nations’ Sustainable Development Goal Target 3.5, which aims to strengthen the prevention and treatment of substance abuse, including narcotic drug abuse and the harmful use of alcohol.

## 1. Introduction

Substance use/abuse remains a public health concern worldwide, affecting society on every level with variations in life stages, types, prevalence, and risk factors [1,2,3]. The Centers for Disease Control and Prevention (CDC) refers to substance use as the use of selected substances injected or otherwise absorbed into the body with possible dependence and other detrimental effects [4]. These substances include alcohol (beer, wine, and distilled liquors) and tobacco (cigarettes, vapor-cigarettes, cigars, chewing tobacco, and snuff), which are legal, as well as illegal cannabinoids (marijuana/cannabis/dagga and edible cannabinoids), opioids, cocaine, and hallucinogens. Substance use affects young people aged 10–24 years [5], and the age of onset for substance use is estimated between 13 and 15 years in low- and middle-income countries (LMICs) and under 20 years in Europe, Australia, and North America [6]. Globally, 53% of people aged 15 years and above have ever used substances (mainly alcohol) [1,7]. In sub-Saharan Africa (SSA), 42% of adolescents and young adults (AYAs) use substances, while 33% use alcohol, followed by tobacco use (25%) and cannabis (16%) [1,2]. In the Southern African region, 37% of AYAs use substances, such as alcohol (41%), tobacco (46%), and cannabis (26%) [2]. While, in South Africa, alcohol (22–66%) and tobacco (46%) are the most used substances among school AYAs [3,8,9,10,11], the use of cannabis and other substances has also been documented [12,13,14].

Substance use burdens the educational and social systems and has health and behavioral implications [1,2,7]. The curiosity of AYAs to experiment with substances leads to addiction/dependency [1,10,11,12], and predisposes them to sexual risk behaviors [13,14,15], respiratory conditions, oral pathologies, and decreased physical capacity [15,16]. Others reported negative influences on mental health including psychosocial problems (anxiety and depressive symptoms) and psychological distress [16,17] such as social anxiety, developing psychosis (i.e., hallucinations and paranoia), and schizophrenia (a type of mental illness where people might see or hear things that are not there) [17,18]. Substance use is triggered by wanting to feel good, feel better, and do better, out of curiosity or because others are doing it (i.e., social influence) [9,19]. Among AYAs, reasons are based on seeking new experiences and taking risks, sharing a social experience, or feeling part of a social group, relieving stress, and relieving symptoms of mental health disorders such as anxiety and depression [9,20]. Risk factors associated with substance use are multifaceted, encompassing various domains. These include experiences of childhood trauma [21] as well as demographic and socioeconomic variables [22,23]. Moreover, factors related to educational attainment, engagement in extracurricular activities or part-time employment, financial autonomy, and receiving a childcare grant also play contributory roles [3]. Additionally, psychological attributes such as poor self-control, inadequate parental supervision, parental attitudes, social networks, peer influence, and the presence of certain mental health conditions such as attention-deficit/hyperactivity disorder (ADHD) are implicated [20]. Notably, these risk factors have been observed across multiple African [24,25,26] and South African [9,27,28] contexts, highlighting their broad relevance and potential impact on substance use behaviors.

Studies in South Africa have reported variations in the prevalence of substance use, types, reasons, related effects and complications, and contributing factors in various provinces/regions [3,9,14,16,27,29,30,31,32,33] including the South Africa National Youth Risk Behavior Survey (SANYRBS) [34]. Substance use has reportedly reached epidemic proportions among high school AYAs however, the disproportionality of minimal research by provinces is evident and calls for continuous and translational research, bearing in mind that South Africa has ethnicity and cultural diversity, and pockets of poverty [35]. In South Africa, AYAs are confronted with diverse and context-based social [3,9,36] and health issues [37,38] amidst the burden of disease [39,40]. To date, the SANYRBS remains a comprehensive national baseline survey in South Africa since the implementation of the compulsory education system and contributes significantly to informing the evidence base for future health promotion intervention planning [41], although with limited data on AYAs’ perceptions of substance use and risk factors emerging recently in under-resourced settings in South Africa. One such intervention is the implementation of comprehensive school-based prevention programs that incorporate elements such as education on the risks of substance use, skill-building to resist peer pressure, and fostering positive social norms regarding substance use. Given no tangible progress in reducing substance use among school learners in South Africa, we conducted a cross-sectional quantitative study to investigate substance use among AYAs in public high schools in Mpumalanga, South Africa. If care is not taken, substance use among young people seems likely to persist as one of the public health burdens with implications on health and behavior, as well as burdening the educational and social systems [1]. We envisaged that continuous translational research such as this will add knowledge and inform evidence-based multilevel–multicomponent interventions to minimize the abuse and effects of substances in schools in alignment with the United Nations’ Sustainable Development Goals (SDG) target 3.5. The SDG Target 3.5 aims to strengthen the prevention and treatment of substance abuse, including narcotic drug abuse and harmful use of alcohol. This target is part of the broader SDG 3, which seeks to ensure healthy lives and promote well-being for all at all ages [42].

Moreover, the implementation of school policies and environmental changes can also contribute to substance abuse prevention efforts. This may include enforcing strict policies regarding substance use on school premises, promoting alternative recreational activities, and fostering a supportive and drug-free school environment. Since the legislative frameworks related to substance use differ from country to country, in South Africa, there are age limits for purchasing alcohol and tobacco products, typically ranging from 18 to 21 years old. This is the same as the legality of cannabis, with some countries legalizing its use for medicinal or recreational purposes, while others maintain strict prohibition laws around its use.

**Figure 1:**
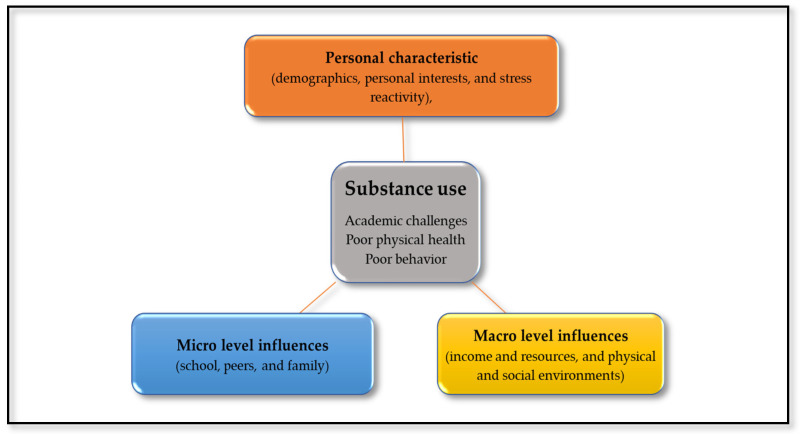
Adapted Socio-Ecological Model on factors influencing adolescent substance use.

Generally, the Socio-Ecological Model provides a comprehensive approach to understanding and addressing the multiple levels of influence on health behaviors and outcomes, and it informs the development of multilevel interventions to promote health and prevent disease [43,44]. Theoretically, risk factors of substance use among AYAs are grouped into three broad categories: personal level influence (genetic susceptibilities, mental health and personality traits, neurological development, and stress reactivity), micro level influences (family influences, school influences, and peer influences) and macro level influences (income and resources, social environment, and physical environment), as reported by the United Nations Office on Drugs and Crime Prevention (UNODC) [45]. The conceptual framework related to the factors investigated in this study is comprehensively presented in Figure 1.

## 2. Methods

### 2.1. Study Design, Setting, and Population

This is a cross-sectional and quantitative study conducted between August 2022 and January 2023. The study was conducted in the Dr. JS Moroka municipality situated in Mpumalanga province, South Africa. While several studies have been conducted in other provinces such as Gauteng and Free State Western Cape, Mpumalanga remains less studied regarding substance use and other social issues. We randomly selected the Dr. JS Moroka municipality as the study setting.

Mpumalanga province is found in the north-eastern part of South Africa and is bordered by four out of nine provinces, which are Gauteng, Limpopo, Free State, and KwaZulu-Natal [46]. The Dr. JS Moroka municipality in the Nkangala district is inhabited by 99% black Africans with serious challenges in terms of unemployment, poverty, and inequality. The poverty headcount ratio in South Africa’s national poverty line has been estimated at 56% in 2020 and increased from 40% in 2011 [47]. The gap between the rich and the poor has widened due to unemployment resulting in the income gap, with the wealthiest 20% of South Africans earning almost 68 times more than the poorest 20%. This disparity has serious consequences for social cohesion, as it causes continuous inequality and hinders economic growth [47].

### 2.2. Sample Size and Sampling Procedure

Using the Cochran formula and taking into consideration an estimated total enrolment figure of approximately 25,000 high school AYAs in this municipality, a 95% confidence interval, and a 5% margin of error, a minimum representative sample was calculated using the Raosoft sample calculator. The calculated minimum representative sample size was 379 participants. Buffered with 10% for possible incomplete or missing data, the total sample size for the study will be 412.

A multi-stage sampling technique was used to select schools, learning grades, classes, and AYAs. First, high schools were selected randomly, and out of the 12 (twelve) schools situated at the Mametlhake and Nokaneng education circuits within the Dr. JS Moroka municipality, four (4) schools were selected. Second, within the selected schools, a simple random sampling of learning grades, classes, and learners was carried out. High schools used in this study previously belonged to the former Bophuthatswana homeland in South Africa and they were called high schools starting with Grade 10 (previously called standard 8), Grade 11 (previously called standard 9), and up to Grade 12 (previously called standard 10). Enrolment numbers by school were as follows: first school (S1) ≈ 659, second school (S2) ≈ 993, third school (S3) ≈ 400, and fourth school (S4) ≈ 555. The sampling plan entailed treating each school as a unit of analysis with a sample size between 90 and 150 to avoid disproportionate sampling. However, a lower sample was obtained in one school due to the reluctance of parents to consent to the participation of their dependencies.

AYAs were recruited during school visits and the principals of schools were provided with letters addressed to the school governing bodies, an ethical certificate from the Sefako Makgatho Health Sciences University Research and Ethics Committee (SMUREC), and a permission letter from the Mpumalanga Department of Education to access high schools in the Mametlhake and Nokaneng education circuits. After obtaining permission to conduct the study in the selected high schools, the research team further liaised with the allocated teacher to facilitate obtaining parental consent for their dependents and identifying AYAs from the grades and classes. Selected AYAs were engaged in the procedure of the study and preparations for data collection.

### 2.3. Data Collection and Tools

A pre-tested structured self-administered questionnaire was used to collect data on socio-demographic data and substance use. The questionnaire was adapted from similar studies conducted on substance use among school adolescents in South Africa, including the SAYRBS [32,33,48,49]. Data were collected on the demographic data of AYAs such as learning grade, gender, and age, and previous grade performance. Demographic characteristics of participants obtained included, age, sex, school, learning grade, participation in recreational/sports, and religious activities, while their household socio-economic status obtained included parents’/guardians’ marital status, education level, employment status, and household infrastructure. Information on substance use included past and current use (in the last 12 months), age of first use, types, as well as availability, reasons, and related effects. According to the WHO, alcohol consumption in the past 12 months is defined as the proportion of adults (15+ years) in a given population who have consumed any alcohol during the past 12 months, assessed at a given point in time [50].

Before data collection, content, face, and construct validity were checked to ensure the quality of the questionnaire. In content validity, experts in the field ensured the adequacy of the questionnaire to measure the intended constructs [34], while face validity was based on the layout of the questionnaire. The questionnaire was translated from English to a local language (i.e., Setswana) by an independent translator who is conversant in the two languages. A pilot study was conducted to determine the feasibility of the main study. During the pilot study, conducted in one high school that was not part of the main study, research assistants were trained and assessed while assisting the AYAs in filling out the questionnaire. After pretesting the questionnaire, adjustments such as minimal clarity of wording and simplifying the layout and style were performed on the questionnaire. The results from a pilot study informed the feasibility of statistical analysis to ascertain the determinants of substance use and did not form part of the main study.

Thereafter, selected AYAs with parental consent were arranged to gather in their respective schools on the day of data collection. The research team engaged AYAs on the purpose and the procedure of the study, and they were given the opportunity to ask questions. AYAs who were 18 years and above were given consent forms, and those who were below 18 years and had parental consent were requested to give assent to participate in the study. Questionnaires were distributed to adolescents to complete in the presence of the first author and the two research assistants to give assistance when necessary and lasted for 20 to 30 min. During data collection, there were no interruptions to the daily running of the schools, and engagements with adolescents were conducted after school hours. On a rare occasion, we used weekends and school holidays, as per the agreement with parents, permission from the SGB, and ethical clearance from the Mpumalanga Provincial Department of Education (South Africa), with approved timelines to visit all four high schools.

### 2.4. Statistical Analysis

Data were analyzed using STATA (Intercooled Stata^®^ Version 18). Complete case analysis (CRA) was used to identify participants with missing data during analysis. After checking for data distribution using the Skewness–Kurtosis test for numerical data, descriptive and inferential statistics were computed. Descriptive statistics were used to analyze data on substance use, types, reasons for use, related effects, and risk factors, as well as adolescents’ demographics and households’ socio-demographics (%). Chi-square/Fisher’s exact test was used to compare differences in proportion between two categorical groups. Results are presented as frequencies (n) and percentages (%), Chi-square (χ2) and *p*-value. Bivariate and multivariable-adjusted logistic regression analyses were used to assess the risk factors of substance use based on factors associated with substance use in the bivariate analysis at *p* < 0.20. Following that, a stepwise backward elimination procedure was used to eliminate confounders and only factors associated with substance use at *p* < 0.05 were retained in the final model. Results are presented as crude odds ratios (OR) and adjusted odds ratios (AOR) with a 95% confidence interval (CI) and for all analyses, statistical significance was considered at *p* < 0.05.

### 2.5. Ethics Statement

This study was conducted according to the guidelines laid down in the Declaration of Helsinki, and all procedures involving human subjects were approved by the Sefako Makgatho Health Sciences University Research and Ethics Committee (SMUREC) (SMUREC/H/137/2022: PG). Further permission was sought from the Mpumalanga Provincial Department of Education (South Africa) to access schools in the municipality. Following that, permission was sought from the schools’ principals and governing bodies of the selected high schools. Post receiving permission from the schools, written consent was requested from the parents before engaging students, as described in the recruitment of learners. Additionally, during data collection, written informed consent was obtained from adolescents who were aged 18 years, and assent from adolescents who were aged below 18 years.

## 3. Results

### 3.1. Prevalence of Substance Use and the Types of Substances

Ten questionnaires had missing data of over 10%, especially on the primary outcome, and were excluded during data analysis. The final sample of 402 AYAs was obtained, and their mean age was 18 ± 1 years ranging from 14 to 23 years. Almost half (45%, 95%CI: 40–50%) of them reported using one or more substances in the last twelve months, and most, 92% (95%CI: 87–95%), started using substances before the age of 18 years. Alcohol was the most used substance (74%), followed by cigarette (12%) and cannabis (11%) use, and other substances such as nyaope (i.e., a form of black tar heroin, sometimes mixed with other substances). Results are presented as frequencies (N) and proportions (%) in Table 1.

### 3.2. Demographic Characteristics of Adolescents and Guardians

In Table 2, the Chi-square test was used to compare demographic characteristics between AYAs who use substances and the non-users. The sample included 162 (40%) boys and 240 (60%) girls. Adolescents were divided into two groups: younger (<18 years) and older (≥18 years). Sample sizes by schools were 93 (23%) in the first school (S1); 133 (33%) in the second school (S2); 131 (33%) in the third school (S3); and 45 (11%) in school number 4 (S4) with the lowest participation of adolescents. AYAs were distributed across grade 10 (n = 90), grade 11 (n = 85), and grade 12 (n = 227). Only 13% of AYAs reported participation in both recreational activities/sports and religious activities. Significant differences of substance use were observed by school (*p* ≤ 0.0001) and participation in recreational/sports activities (*p* ≤ 0.0001).

Chi-square/Fisher’s exact tests showed significant differences of substance use with parents’/guardians’ marital status, household size, water access, electricity use, and type of toilet. Most parents/guardians of AYAs had grade 12 education, were married (84%), unemployed (52%), lived in larger households (85%), and with a monthly income below R5000 (85%). Poor sanitation and infrastructure were observed by the use of pit toilets (95%), while electricity and water access were reported in 90% of households in Table 3.

### 3.3. Availability, Reasons, and Related Effects of Substance Use

Table 4 shows availability, reasons, and related effects of substance use. Over two-thirds of AYAs reported availability of alcohol outlets near schools (70%) and in the communities (70%). Substance use among AYAs was common at social events (i.e., parties/social occasions) (47%), streets and homes (43%), and less common at the recreational/sports platforms (10%) with minimal participation in sports/recreational activities (13%). About two-thirds of AYAs (60%) were aware of substance use complications, and at some point, while using substances, they reported experiencing excitement/euphoria (28%), nausea (17%), aggressiveness (16%), lack of respect (15%), and lack of studying (10%). Also, at the time of the study, AYAs reported episodes of hallucinations, sleeping disorders, body weakness, and dry mouths due to substance use.

### 3.4. Risk Factors for Substance Use

Table 5 shows the association of substance use and risk factors. In univariable logistic regressions (crude odds ratio (COR)), substance use was associated with age, attending a particular high school, learning grade, participation in recreational/sports activities, and parents’/guardians’ marital status, and household size (*p* ≤ 0.25). After controlling for potential confounders (i.e., age, sex, age of first use, and guardians’ employment status and household income), the final hierarchical logistic regression showed significant associations of substance use with attending a particular high school (S4) (AOR = 3.93, 95%CI: 1.72–8.99), learning grade 12 (AOR = 2.73, 95%CI: 1.46–5.11), substance availability in communities (AOR = 22.45, 95%CI: 2.75–183.56), participating in recreational/sports activities (AOR = 9.74, 95%CI: 4.21–22.52), and living in larger households (AOR = 2.96, 95%CI:1.57–5.58).

## 4. Discussion

The concern about the ongoing substance use among young people accompanied by negative social and health outcomes can never be overemphasized. For this reason, we investigated substance use among school adolescents and young adults (AYAs) in rural public high schools in the Dr. JS Moroka municipality in Mpumalanga, South Africa. Almost half of AYAs in this study reported current use of a substance, like reports in other studies [3,9,14,16,27]. The prevalence of substance use among AYAs varies in SSA [2,51], attributed to several factors including socio-environmental, liberal attitudes and practices, social norms, lack of effective substance enforcement, lack of effective implementation of laws, as well as access and availability of these substances [2,51]. AYAs who participated in the current study started substance use while they were below 18 years of age [52,53], which has previously been reported in South Africa, despite the National Liquor Act prohibiting alcohol sale to individuals younger than 18 years of age [9,54]. Initiation of substance use in early adolescence age (age ≤ 13 years) has serious public health implications and consequences such as poor performance at school, dropout, developing alcohol addiction/dependence, experiencing mental and social harm, and cardiovascular diseases later in adulthood [55,56]. Our results show that 45% of school AYAs were currently using substances at the time of the study, suggesting an ongoing public health problem predisposing them to social, behavioral, and health issues.

Over two-thirds of AYAs reported alcohol (beer, cider, and wine) as a common substance used, followed by cigarette smoking and cannabis. According to the WHO [57], beer, wine, and spirits are mostly consumed in SSA and vary by geographical differences and population groups. In South Africa [3,9,14,27,31,32,58], alcohol use is common, the same as in most SSA countries [2,51,59], Europe [60], and the USA [61,62]. Cigarette smoking and cannabis were less likely used after alcohol use, same as in previous South African studies [14,28,31,63,64] and other countries like Ethiopia [65] and Ghana [66]. Furthermore, the availability of alcohol outlets near most schools and the presence of various substances in the communities have been reported in our study, like in other local studies [58,67,68] and in other countries [69,70,71]. Also, easy access to alcohol, cigarette, and cannabis substances, also observed in this study, has been implicated as the main contributing factor to the use of various types of substances [14,31,32]. Stringent measures to halt the availability of substances around schools and communities, as well as easy access, should be put in place. Such measures that could be implemented may include firstly, community policing and surveillance by involving regular patrols, targeted operations, and collaboration with local authorities to identify and address areas of high substance availability. Secondly, community engagement and awareness campaigns by fostering community involvement in substance abuse prevention efforts through educational campaigns, community forums, and neighborhood watch programs. Thirdly, supporting alternative outdoor activities that provide opportunities for AYAs to participate in positive recreational, cultural, and educational activities as alternatives to substance use. This could include expanding access to sports programs, arts and music classes, after-school clubs, and community events that promote healthy lifestyles and social connections.

The current study further showed that AYAs predominantly engaged in substance use during social events, on the streets, and in their homes, with fewer instances reported at recreational or sports platforms. This highlights the significant role of environmental contexts in shaping substance use behaviors within this demographic. These findings align with existing studies that underscore the impact of social settings on AYAs’ substance use [72,73,74]. Social events, streets, and homes often serve as venues for social interactions and peer influences, contributing to the increased likelihood of substance use initiation and engagement in these settings [43,75]. The prevalence of substance use at social events indicates the influence of peer dynamics and socialization processes on AYAs’ substance use behaviors. Peer influence has been identified as a potent factor in the initiation and maintenance of substance use during adolescence [43,76]. The prominence of substance use on streets and in homes further emphasizes the significance of familial and community environments in influencing AYAs’ engagement with substances. Family dynamics, including parental attitudes and behaviors, may contribute to the normalization or acceptance of substance use within the home environment, thereby influencing adolescents’ perceptions and behaviors [77,78]. Therefore, the availability and accessibility of substances on streets may expose AYAs to increased opportunities for experimentation.

Similarly, the comparatively higher likelihood of substance use at recreational/sports platforms suggests that these settings may be conducive to substance use among AYAs. Researchers have highlighted the influence of recreational habits, particularly in the weekend night-life context, on drug use [79]. Veliz and colleagues [80] also emphasized that the type of sports participation can influence substance use, with high-contact sports associated with higher odds of substance use. Nevertheless, recreational and sports activities often provide structured and supervised environments, which may act as protective factors against substance use initiation [72,73]. As such, we believe that the presence of positive role models, organized activities, and a focus on skill development in these settings may help to reduce the likelihood of engaging in substance use in this community.

Reasons/motivations for substance use were diverse among AYAs and cited as seeking joy, curiosity, eliminating stress, and succumbing to social influences from family and friends. These motivational factors align with previous research that has identified similar drivers for substance use in AYAs [81,82]. Researchers have reported that learners are prone to substance use due to various reasons, like academic and peer pressure, the appeal of popularity and identification, readily available pocket money, and relatively easy accessibility of several substances [83,84]. Further, AYAs are vulnerable to substance use either due to peer pressure from their friends and family or because of being lonely. Therefore, this multifaceted nature of motivations underscores the complexity of addressing substance use behaviors within this demographic. However, these behaviors should be discouraged since they may affect the academic performance and physical growth of AYAs, and therefore a need for social resistance training skills for one not to succumb to pressure is required [85]. Therefore, in this setting, it would be important to equip AYAs with skills and strategies to resist peer pressure and make healthy choices regarding substance use. To mention a few examples, school-based prevention programs could integrate substance use prevention modules into the school curriculum, focusing on building refusal skills, assertiveness, and decision-making abilities. Secondly, peer support groups could be established within schools or communities where AYAs can connect with peers who share similar values and goals regarding substance use avoidance. Peer mentors can provide guidance, support, and encouragement to resist peer pressure and make positive choices.

Regarding risk factors, substance use was significantly associated with attending a particular high school, being in learning grade 12, substance availability in communities, participating in recreational/sports activities, living in larger households, as well as several socio-demographic variables such as parents’/guardians’ marital status and infrastructure and sanitation issues on bivariate analysis. These findings are similar to other studies in South Africa [3,27,31,58,86] and other countries [85,87]. However, inconsistent determinants of substance use among AYAs by age, sex, setting, learning grade, and school attended have been reported [3,27,53,88], mostly attributed to the differences in alcohol drinking patterns [27]. In this case, AYAs who are in grade 12 were likely to use substances in this study, compared to those in grades 10 and 11. According to the Youth Research Unit (YRU), most learners who use drugs are in grade 12, and this is attributed to peer pressure and the desire to be socially accepted amidst stress relief and recreational purposes [89].

Episodes of lack of studying, lack of respect, and aggressiveness were reported in this study. In particular, substance use has been associated with aggression, influencing the course and risks of substance-use disorders among AYAs [90]. Previous research has reported that AYAs who use substances encounter social harms, such as physical fights and injury/accidents [9] in addition to repeating a grade, absenteeism, missing school, dropout, and low academic performance [3,91,92,93,94]. Some AYAs reported experiencing complications with hallucinations, sleeping disorders, body weakness, and dry mouths, which reveals the need for understanding of the impact of substance use on the physical and mental well-being of this population. This is because hallucinations are perceptual disturbances that can significantly impair cognitive function and reality perception [95], such that previous studies have linked hallucinatory experiences to substance use, particularly in the context of hallucinogenic substances [96,97]. The reported complications in this study align with the existing literature that underscores the potential psychological repercussions of substance use among AYAs [95,96,97,98]. Our study further noted sleeping disorders as another complication. Disruptions in sleep patterns can have detrimental effects on overall health and well-being, particularly in a developmental stage where adequate sleep is crucial [99]. Substance use has been consistently associated with sleep disturbances among adolescents and young adults [100,101]. As such, our findings reinforce the existing literature by highlighting the prevalence of sleeping disorders as a potential consequence of substance use among AYAs.

Over two decades ago, the South African government intended to secure schools as safe and disciplined learning environments to enhance quality education, and as a result, published the National Policy of Drug Abuse Management in Schools guidelines to manage substance use in school environments [39]. To date, no progress related to this policy to address substance use has been recorded, as attested by colleagues [39]. There are several successful school-based programs reported in Australia (School Health and Alcohol Harm Reduction Project (SHAHRP)) [102], the United States of America (Project ALERT [103] and ALERT Plus [104]), and Europe (the Unplugged program) [105]. These programs have shown promising results in enhancing the reduction of substance use among youth [106], mainly because they incorporate skills training aimed at changing attitudes, promoting social and emotional abilities, critical thinking, and problem-solving compared to traditional intervention programs focusing on changing attitudes and perceptions on substance use and increasing awareness about the negative effects of peer pressure [106]. Therefore, these programs might also be relevant in our context considering that AYAs are an important target group for substance use prevention and interventions and intensified efforts to address this crisis must be on a school level.

Our results should be considered with some limitations. The cross-sectional design used allowed for reporting on the inferences rather than causality. The use of four high schools in the area, although selected randomly, limits generalizing the results, mainly because the study was limited to black AYAs in rural high schools. We did not control for the number of different high schools as the unit of analysis, and as a result, did not account for the potential clustering effect, where students from the same high school are likely to be more like each other compared to students from different schools. This clustering can lead to alpha inflation, making the results appear more significant than they might be if the school-level variance were properly controlled. In addition to prevalence, types, and reasons/motivations, this study reports on proximal determinants of substance use rather than distal determinants, which could have been elaborated on based on the pricing and marketing of substances. Social desirability, response, and recall biases might have been introduced during data collection originating from AYAs self-reporting substance use, types, and reasons. Most importantly, although we validated a tool used in this study and tested for reliability, the use of standardized tools such as the Alcohol Use Disorders Identification Test could have provided conclusive results and must be adopted in future studies. However, the prevalence, types, reasons, and risk factors correspond with local studies, although with varying magnitudes and intensities of relationships. Finally, this study did not investigate how and where AYAs obtain substances, and this information could provide insights into effective enforcement strategies to curb availability. Understanding the sources of substances can inform targeted interventions to disrupt supply chains and prevent access to illicit drugs. To address this gap, future research could incorporate qualitative methods such as interviews or focus groups to explore AYAs’ experiences with obtaining substances, including their interactions with dealers, peers, or online platforms. Additionally, collaborating with law enforcement agencies to gather data on drug trafficking patterns and areas of high substance availability could further inform enforcement efforts. By elucidating the pathways through which AYAs access substances, policymakers and stakeholders can develop more targeted and effective strategies to reduce availability and mitigate the harms associated with substance use among this vulnerable population. The use of frameworks emphasizes that individuals are embedded within multiple systems and that the interaction between these systems influences their health and well-being.

## 5. Conclusions

This study highlights current substance use among school AYAs. Alcohol was the most common substance used, followed by cigarette and cannabis use. Availability of alcohol outlets near their schools and various substances in the communities were reported. Further results showed that AYAs used substances out of social influence, curiosity, to find joy, and eliminate stress, especially in social events, on the streets, and at home. They also reported experiencing negative physical health outcomes due to substance use, mainly hallucinations, sleeping disorders, body weakness, and dry mouths. In the final model, risk factors for substance use were attending a particular high school, being in grade 12, availability in communities, participating in recreational/sports activities, and living in larger households. These findings advocate for prevention and intervention efforts that should consider these specific health concerns to develop targeted strategies for mitigating substance use and its adverse consequences in this vulnerable population.

Our conclusion underscores the importance of tailored prevention and intervention initiatives to address the specific health challenges faced by HIV-positive individuals with substance use disorders, to achieve the United Nations’ Sustainable Development Goals (SDGs) Target 3.5. SDG Target 3.5 aims to strengthen the prevention and treatment of substance abuse, including narcotic drug abuse and harmful use of alcohol. To effectively curb substance use in the municipality where the high schools are located, the first step should involve conducting a comprehensive needs assessment and situational analysis to understand the specific factors contributing to substance use among learners. This assessment should involve collaboration with local stakeholders, including schools, healthcare providers, law enforcement agencies, and community organizations. Additionally, it is crucial to prioritize evidence-based prevention strategies such as education and awareness campaigns, peer support programs, access to mental health and substance use services, and enforcement of policies to restrict access to substances.

Adapting some of the programs mentioned previously, such as the SHAHRP [102], Project ALERT [103], ALERT Plus [104], and the Unplugged program [105], might also assist in our case due to the reported promising results in enhancing the reduction of substance use among youth [106], and the incorporation of skills-training to change attitudes, promote social and emotional abilities, critical thinking, and problem-solving [106].

## Figures and Tables

**Table 1:** Prevalence of substance use and the types.

Variables	Categories	n	%
Past substance use	No	222	55
	Yes	180	45
Starting age for substance use	<18 years	166	92
	≥18 years	14	8
Current substance use	Non-users	222	55
	Users	180	45
Types of current substance use (of current users)	Alcohol	134	74
	Cigarette smoking	22	12
	Cannabis	19	11
	Other	5	3
Frequency of substance use	1–2 times/week	53	29
	≥3 times/week	130	72

Past substance use means using the substance previously at some point in their life, but not currently. Current substance use means using the substance at the time of the study.

**Table 2:** Comparison of demographics among substance non-users and users.

Variables	All N (%)	Non-Users N (%)	Users N (%)	*χ*2	*p*-Value
Participant’s high school				20.95	≤0.0001
S1	93 (23)	56 (25)	37 (21)		
S2	133 (33)	34 (15)	11 (6)		
S3	131 (33)	78 (35)	53 (29)		
S4	45 (11)	54 (25)	79 (44)		
Sex				0.009	0.925
Boys	162 (40)	133 (60)	107 (60)		
Girls	240 (60)	89 (40)	73 (40)		
Age group (years)				0.089	0.767
Younger (<18)	173 (43)	97 (44)	76 (42)		
Older (≥18)	229 (57)	125 (57)	104 (58)		
Learning grade				1.642	0.440
Grade 10	90 (22)	55 (25)	35 (19)		
Grade 11	85 (51)	45 (20)	40 (22)		
Grade 12	227 (56)	122 (55)	105 (58)		
Participating in recreational/sports					≤0.0001
No	351 (87)	211 (95)	140 (78)		
Yes	51 (13)	11 (5)	40 (22)	26.76	
Participating in religious activities					0.801
No	351 (87)	29 (13)	22 (12)		
Yes	51 (13)	193 (87)	158 (88)	0.063	

**Table 3:** Comparison of guardians’ socio-demographics among substance non-users and users.

Variables	All N (%)	Non-Users N (%)	Users N (%)	*χ*2	*p*-Value
Married				16.65	≤0.0001
No	63 (17)	20 (9)	43 (24)		
Yes	339 (84)	222 (91)	137 (76)		
Education level				5.521	0.137
Primary education	35 (9)	14 (6)	21 (12)		
High school	74 (18)	44 (20)	30 (17)		
Matric	189 (47)	111 (50)	78 (43)		
Post matric	104 (26)	23 (24)	51 (28)		
Employment status				0.411	0.814
Unemployed	210 (52)	119 (54)	91 (51)		
Employed	165 (41)	89 (40)	76 (42)		
Pensioner	27 (7)	14 (6)	13 (7)		
Type of house				0.081	0.776
Non-brick	100 (25)	54 (24)	46 (26)		
Brick	302 (75)	168 (76)	134 (74)		
Household size				15.64	≤0.0001
<5 members	340 (85)	202 (91)	138 (76)		
≥5 members	62 (15)	20 (9)	42 (23)		
Household income/month				0.387	0.534
<R5000	340 (85)	190 (86)	150 (83)		
≥R5000	62 (15)	32 (14)	30 (17)		
Electricity				8.224	0.004
No	39 (10)	30 (14)	9 (5)		
Yes	363 (90)	189 (86)	171 (95)		
Water access				8.224	0.004
No	39 (10)	30 (14)	9 (5)		
Yes	363 (90)	189 (86)	171 (95)		
Type of a toilet				24.17	≤0.0001 *
Pit	380 (95)	221 (99)	159 (83)		
Flush	22 (5)	1 (1)	21 (12)		

* NB: Fisher’s exact test.

**Table 4:** Availability, reasons, and related effects of substance use.

Variables	Categories	N	%
Availability of substance outlets near schools	No	119	30
	Yes	283	70
Availability of substance in communities	No	24	6
	Yes	378	94
Places where you used substance	Parties/occasions	85	47
	Home/streets	77	43
	Recreation/sports platforms	18	10
Reasons/motivation for substance use	Joy seeking	75	42
	Teenage curiosity	39	22
	Eliminate stress.	26	14
	Social influence (peers/family)	18	10
	Lack of recreational facilities	10	6
	Other	12	7
Aware of related effect	No	158	39
	Yes	242	60
Experienced effects of substance use	Excitement/Euphoria	51	28
	Nausea	31	17
	Aggressiveness	29	16
	Lack of respect	27	15
	Lack of studying	18	10
	Other	24	13
Experienced physical health outcomes	Hallucinations	86	48
	Sleeping disorders	41	23
	Body weakness	25	14
	Dry mouths	28	16

**Table 5:** Risk factors for substance use.

Current Substance Use	COR (95%CI)	*p*	AOR (95%CI)	*p*
Schools				
S1	1 (ref)		1 (ref)	
S2	2.04 (0.92–4.53)	0.079	1.48 (0.63–3.48)	0.365
S3	2.10 (0.98–4.51)	0.057	1.62 (0.71–3.70)	0.253
S4	4.52 (2.11–9.70)	≤0.0001	3.93 (1.72– 8.99)	0.001
Learning grades				
Grade 10	1(ref)		1 (ref)	
Grade 11	1.43 (0.78–2.61)	0.246	1.97 (0.97–4.02)	0.062
Grade 12	1.34 (0.82–2.21)	0.247	2.73 (1.46–5.11)	0.002
Availability in communities				
No	1 (ref)		1 (ref)	
Yes	20.69 (2.77–154.75)	0.003	22.45 (2.75–183.56)	0.004
Participation in recreational/sports activities				
No	1 (ref)		1 (ref)	
Yes	5.48 (2.72–11.04)	≤0.0001	9.46 (4.12–21.70)	≤0.0001
Household size				
<5 members	1 (ref)		1 (ref)	
≥5 members	3.07 (1.73–5.46)	≤0.0001	2.96 (1.57–5.58)	0.001

## Data Availability

The data that support the findings of this study are available from the corresponding author upon reasonable request.
